# Risk, Resilience, and Timing: The Impact of War on Later-Life Pain

**DOI:** 10.1093/geroni/igaf122.073

**Published:** 2025-12-31

**Authors:** Rui Huang, Yuhang Li, Hanna Grol-Prokopczyk

**Affiliations:** University at Buffalo, Buffalo, New York, United States; University at Albany, Albany, New York, United States; University at Buffalo, Buffalo, New York, United States

## Abstract

Life course theories predict that early-life war experiences will impact later-life health through both risk and resilience processes, which may vary by age cohorts. However, limited research has empirically examined the resilience process following war and the heterogeneous pathways across age cohorts. This study uses data on older adults from the 2018 Vietnam Health and Aging Study (N = 1,826) to explore associations between early-life war experiences and later-life moderate/severe chronic pain, highlighting the role of war-related risk or protective factors (socioeconomic status, health behaviors, social engagement, and health conditions), and testing for heterogeneity of war impacts between child (aged 6-11), adolescent (aged 12-17), and young adult cohorts (aged 18-23) during the wartime (1965). Regression models reveal that Vietnam War exposures dramatically increase the risk of later-life pain, and that war generates both risk factors (e.g., lower educational attainment, higher smoking risk, greater physical disease count, larger distress, and PTSD score) and protective factors (e.g., greater social engagement). However, KHB mediation analyses show that the impact of war on pain is primarily driven by increases in physical and mental health problems. Children were more vulnerable to the effects of wartime violence on later-life pain than older age groups. As for risk/protective factors generated by war, the child cohort tends to exhibit poorer coping under the effect of war (as indicated by a higher risk of smoking), whereas this group appears to have a better outcome in terms of a greater increase in occupational prestige and less deterioration in physical health.

